# Piezoelectric biosensor with dissipation monitoring enables the analysis of bacterial lytic agent activity

**DOI:** 10.1038/s41598-024-85064-x

**Published:** 2025-01-27

**Authors:** Radka Obořilová, Eliška Kučerová, Tibor Botka, Hana Vaisocherová-Lísalová, Petr Skládal, Zdeněk Farka

**Affiliations:** 1 Department of Biochemistry, Faculty of Science, Masaryk University, Kamenice 5, 625 00 Brno, Czech Republic; 2 Central European Institute of Technology, Masaryk University, Kamenice 5, 625 00 Brno, Czech Republic; 3 Department of Experimental Biology, Section of Genetics and Molecular Biology, Faculty of Science, Masaryk University, Kamenice 5, 625 00 Brno, Czech Republic; 4 FZU – Institute of Physics of the Czech Academy of Sciences, Na Slovance 1999/2, 182 00 Prague, Czech Republic

**Keywords:** Piezoelectric biosensor, Antimicrobial treatment, Phage therapy, Phage-antibiotic synergy, Multidrug-resistant bacteria, *Staphylococcus aureus*, Biophysical methods, Microbiology techniques, Biosensors, Bacteriophages, Phage biology

## Abstract

Antibiotic-resistant strains of *Staphylococcus aureus* pose a significant threat in healthcare, demanding urgent therapeutic solutions. Combining bacteriophages with conventional antibiotics, an innovative approach termed phage-antibiotic synergy, presents a promising treatment avenue. However, to enable new treatment strategies, there is a pressing need for methods to assess their efficacy reliably and rapidly. Here, we introduce a novel approach for real-time monitoring of pathogen lysis dynamics employing the piezoelectric quartz crystal microbalance (QCM) with dissipation (QCM-D) technique. The sensor, a QCM chip modified with the bacterium *S. aureus* RN4220 Δ*tarM*, was utilized to monitor the activity of the enzyme lysostaphin and the phage P68 as model lytic agents. Unlike conventional QCM solely measuring resonance frequency changes, our study demonstrates that dissipation monitoring enables differentiation of bacterial growth and lysis caused by cell-attached lytic agents. Compared to reference turbidimetry measurements, our results reveal distinct alterations in the growth curve of the bacteria adhered to the sensor, characterized by a delayed lag phase. Furthermore, the dissipation signal analysis facilitated the precise real-time monitoring of phage-mediated lysis. Finally, the QCM-D biosensor was employed to evaluate the synergistic effect of subinhibitory concentrations of the antibiotic amoxicillin with the bacteriophage P68, enabling monitoring of the lysis of P68-resistant wild-type strain *S. aureus* RN4220. Our findings suggest that this synergy also impedes the formation of bacterial aggregates, the precursors of biofilm formation. Overall, this method brings new insights into phage-antibiotic synergy, underpinning it as a promising strategy against antibiotic-resistant bacterial strains with broad implications for treatment and prevention.

## Introduction

*Staphylococcus aureus* stands out as a leading cause of life-threatening complications, particularly within healthcare facilities, often culminating in severe, and sometimes even fatal, outcomes^1^. The escalating threat posed by antibiotic-resistant bacteria highlights the importance of exploring new treatment strategies and diagnostic methods. Bacteriophages, commonly referred to as phages, represent a promising alternative to antibiotics in the global fight against antibiotic resistance^2^. Clinical applications of tailored phage therapies have demonstrated efficacy in infection treatment, reducing severe complications, such as amputations or even mortality^3^.

Recent studies have demonstrated that combining phages with subinhibitory antibiotic concentrations can yield superior outcomes compared to individual therapies, a phenomenon termed phage-antibiotic synergy (PAS)^4,5^. This synergy may result from bacterial defense mechanism inefficacy against two different agents or accelerated bacterial population eradication due to the increased phage progeny^6–8^.

Phages, ubiquitously present in various environments, including the human body, interact with and regulate bacterial populations^9^. Particularly, lytic phages are preferred for therapy due to their rapid adaptability, which is crucial for personalized treatments in antibiotic-resistant cases^10^. However, some phages can facilitate horizontal gene transfer through transduction or lysogeny, potentially fostering the emergence of pathogenic strains^11,12^. In addition, the historical oversight of phage therapy caused by the widespread success of antibiotics has led to a lack of relevant studies and regulatory frameworks regarding its clinical applications^13^. Initial research indicated the relative safety of phage therapy; however, further studies are needed^14,15^, making a demand for the development of safe, rapid, and reliable methods of phage testing to underpin their biomedical applications.

Conventional methods of bacterial lysis monitoring rely on microbiological cultivation or techniques assessing changes in cell integrity, such as microscopy and flow cytometry^16^. However, these methods have numerous limitations, including only indirect lysis evidence (e.g., culture turbidity), lack of the ability to determine lytic agent concentration over time, low throughput and sensitivity, and laborious sample preparation via staining that can compromise cell viability^17^. Moreover, some methods, like turbidimetry, are only effective for continuous measurement in planktonic cultures and not in biofilms, commonly encountered in healthcare settings^18^. Thus, advanced instrumental methods are required for accurate elucidation of phage interactions with bacterial populations on biologically relevant surfaces or tissues.

Staphylococcal biofilms are complex microbial communities embedded in a self-produced extracellular matrix^19^. Their occurrence is usually associated with enhanced pathogenicity and resistance to various antimicrobials. The extracellular matrix comprises extracellular DNA (eDNA), polysaccharides, and proteins originating from dead cells. The ratio of these components and the conditions for the biofilm formation are strain-dependent. Biofilm formation is usually triggered in stressful environments, e.g., cold temperatures, high salt concentrations, or the presence of antibiotics^20^. Several phases of biofilm development can be distinguished: initial attachment, multiplication of the bacterial cells, clearance of eDNA, maturation of the biofilm, and finally, its dispersal with the colonization of new niches^21^. Common culture-based methods for biofilm characterization include crystal violet staining of the biofilm biomass and streaking of culture on the specialized agar plates with Congo red dye. Both methods are laborious, and their results are only semiquantitative^22^.

Biosensors, especially those from a label-free family, offer a solution to overcome the drawbacks of the conventional methods. They allow direct monitoring of bacterial behavior on the sensor surface over time with high sensitivity, facilitate controlled addition of lytic agents or removal of metabolites for further assessment, and enable straightforward quantitative evaluation and automation of bacterial lysis monitoring. Sensitive label-free techniques based on optical surface plasmon resonance (SPR)^23^ and piezoelectric quartz crystal microbalance (QCM)^24^ designed for monitoring biochemical events at the sensor surface show great promise. However, these approaches are commonly used for bacteria detection^25,26^ or to study bacterial interactions^27,28^ and only rarely focus on real-time monitoring of bacterial growth^29,30^. In our previous work, we demonstrated the potential of SPR for real-time monitoring of phage-mediated lysis of bacteria on a chip^23^. However, as far as we know, there are no reports on real-time monitoring of bacterial lysis using QCM.

The QCM method, first described in air by Sauerbrey^31^, determines the mass changes on the surface of the quartz crystal by monitoring its resonance frequency shift. Recently adapted for liquid environments, QCM holds significant potential in life sciences, particularly with quartz crystal microbalance with dissipation monitoring (QCM-D), which also assesses surface viscoelastic changes^32^. These effects can relate to bacterial cell adhesion^33^, growth, and biofilm formation, as well as to bacterial lysis. QCM was also demonstrated to enable monitoring of the biofilm formation^34,35^ and studying lytic agent effects on artificially constructed biological membranes, simulating cell surfaces^36^.

**Fig. 1:**
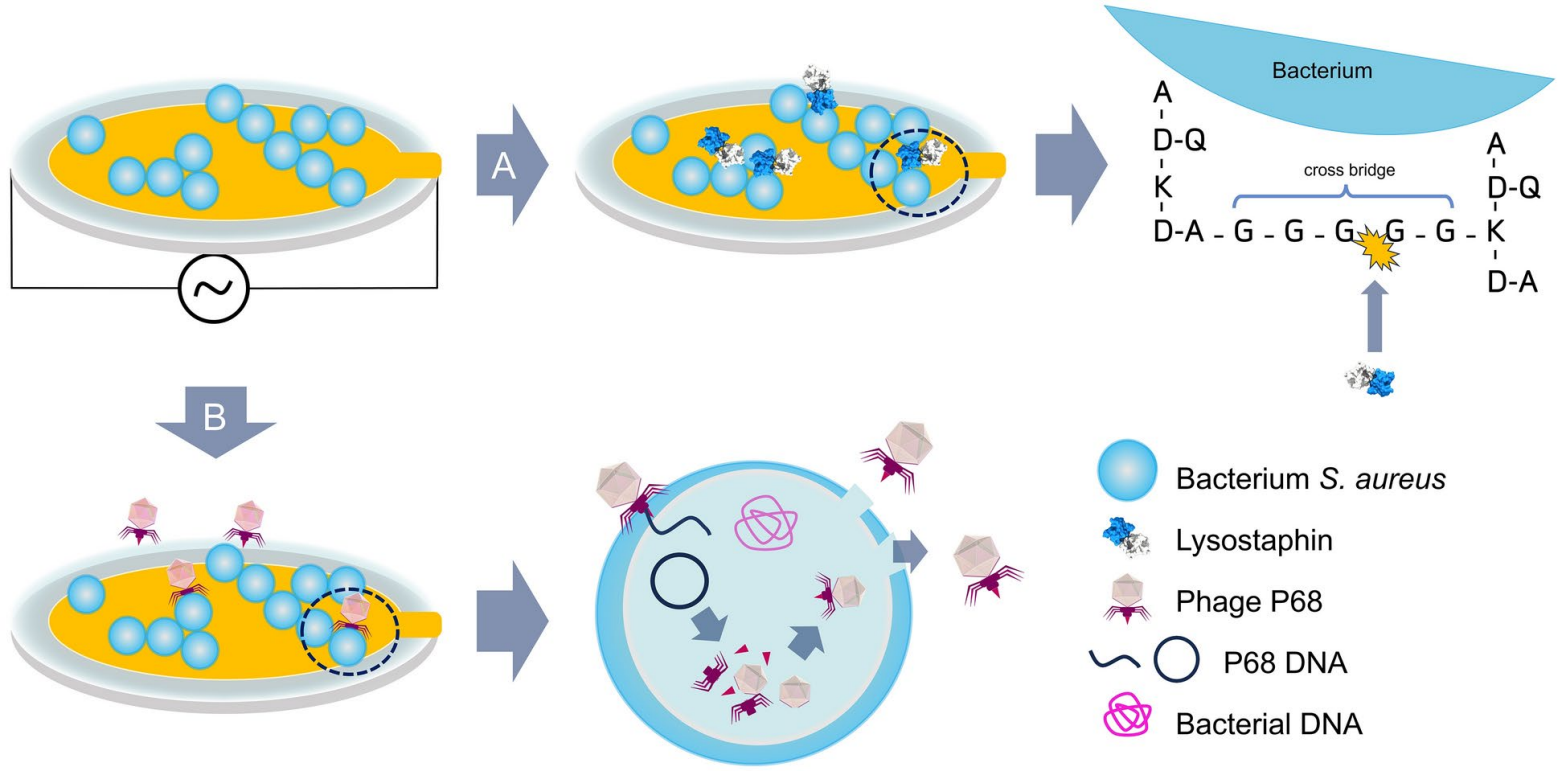
Schematic representation of the course of the experiment on the surface of the QCM-D sensor. (**A**) Monitoring of bacterial lysis by lysostaphin with the detail of the cleavage of the pentaglycine bridge of bacterial peptidoglycan (A – L-alanine, D-Q – D-glutamine, K – L-lysine, D-A – D-alanine, G – L-glycine). (**B**) Phage-mediated lysis on the sensor with the detail of the lytic cycle of the phage in the bacterium.

The aim of the present study was to advance QCM-D to enable real-time and precise monitoring of bacterial lysis in the presence of a complex lytic system, mimicking PAS therapy mechanisms on a chip. To achieve this, we have developed a novel QCM-D methodology using two complex biological systems involving the lysis of *S. aureus* by the enzyme lysostaphin and the phage P68^37^. The approach was based on monitoring changes in resonance frequency and dissipation upon the addition or removal of even a minimal mass caused by the disruption of bacterial integrity and subsequent lysis. Figure 1 illustrates the course of the experiments on the QCM sensor surface, where bacteria were immobilized on a poly-L-lysine (PLL) layer, and the lysis was induced by the enzyme lysostaphin or phage P68. Lysostaphin is an endopeptidase isolated from *Staphylococcus simulans*. In most staphylococcal species, it causes lysis by cleaving the pentaglycine bridges of peptidoglycan^38–40^. Due to its highly specific interaction and precise mechanism of action^41^, lysostaphin can induce rapid lysis of both bacterial cells and biofilms^42^. Phage P68 is a well-characterized podovirus of the *Rountreeviridae* family, known for its short tail and high lytic activity^43,44^. The achieved results demonstrated that studying bacterial lysis on a sensor surface using dissipation monitoring may unveil differences in the formation and eradication of resistant biofilms. Furthermore, the implemented flow system allowed monitoring of metabolites released during the complex bacterium-phage interaction, along with the supply of fresh culture medium. Finally, we employed the developed approach to evaluate the synergistic effect of subinhibitory concentrations of the antibiotic amoxicillin (AMO) and phage P68, enabling the monitoring of lysis in the P68-insensitive *S. aureus* strain.

## Materials and methods

### Chemicals, buffers, and cultivation media

Acetone and isopropanol were purchased from Penta (Czech Republic). Poly-L-lysine (PLL), cysteamine hydrochloride, glutaraldehyde, antibiotic amoxicillin (AMO), and lysostaphin (specific activity ≥ 3990 U/mg) were obtained from Sigma-Aldrich (USA). Growth media were obtained from Oxoid (UK). All other common chemicals were obtained from Sigma-Aldrich (USA), Carl Roth (Germany), or Penta (Czech Republic). Unpolished and polished quartz crystals (resonance frequency of 10 MHz) were purchased from Krystaly, Hradec Králové (Czech Republic). LIVE/DEAD *Bac*Light Bacterial Viability Kit was purchased from Invitrogen (USA).

The used buffers and cultivation media included Tris-buffered saline (TBS; 50 mM Tris, 150 mM NaCl, pH 7.5), phosphate-buffered saline (PBS; 50 mM NaH_2_PO_4_/Na_2_HPO_4_, 150 mM NaCl, pH 7.4), phage buffer (50 mM Tris, 10 mM NaCl, 10 mM CaCl_2_, pH 8.0), and tryptone soya broth (TSB; CM0129, 0.9 g of TSB dissolved in distilled water to 30 mL, pH 7.4).

### Cultivation of *S. aureus*

Four *S. aureus* strains were employed in this study, including two laboratory strains (RN4220 and RN4220 ∆*tarM*) and two clinical strains (13/791 and 10/234). Laboratory bacterial strains *S. aureus* RN4220^45^ and *S. aureus* RN4220 ∆*tarM*^46^ were obtained from Prof. A. Peschel (University of Tübingen, Germany). The deletion of the gene encoding glycosyltransferase TarM, which ensures a precise glycosylation profile of teichoic acid in the bacterial cell wall, makes the RN4220 ∆*tarM* strain highly sensitive to phages from the former *Podoviridae* family, specifically the phage P68 used in this work^46,47^. This phage-bacterium combination results in a very efficient lysis, thus serving as a suitable model. Clinical exfoliative toxin-producing strains *S. aureus* 13/791^48^ and *S. aureus* 10/234^49^ isolated from child blisters were obtained from Dr. P. Petráš (National Institute of Public Health, Czech Republic).

The RN4220 Δ*tarM* strain was selected for the optimization of QCM measurements due to its sensitivity to phage P68, whereas the RN4220 strain served as a control. The following strains were chosen for PAS turbidimetry measurement: RN4220 Δ*tarM* – sensitive to the phage and AMO, RN4220 – insensitive to the phage, sensitive to AMO, 13/791 – sensitive to the phage, resistant to AMO, and 10/234 – resistant to the phage and AMO. Subsequently, the combination of strain RN4220 and phage P68 with AMO was selected for QCM measurement of PAS because of the most pronounced synergistic effect. AMO was selected based on disc diffusion assays with different antibiotics on *S. aureus* strain RN4220 Δ*tarM*, where phage P68 showed enlarged plaques in the presence of AMO (Figure S1A). In addition, phage P68 gained the ability to propagate on strain RN4220 in the presence of AMO (Figure S1B,C). Thus, the chosen phage-antibiotic combination served as a suitable model with unambiguous data interpretation.

The bacteria were grown overnight in TSB medium at 37 °C without agitation to an optical density at 600 nm (*OD*_600_) of ~ 1.8. The cells were centrifuged at 4,600 g for 10 min and resuspended in TBS buffer for efficient immobilization on the sensor surface. The viability of bacteria on the sensor surface after the immobilization was evaluated using fluorescence microscopy. The number of bacteria on the sensor was estimated to be ~ 2.5 × 10^7^ cells, corresponding to approximately 10^8^ colony-forming units (CFU) per mL in a measuring cell with a total volume of ~ 100 μL.

### Preparation of phage P68

The phage P68^37^ (GenBank acc. no. NC_004679.1) was purchased from Félix d’Hérelle Reference Center for Bacterial Viruses (Université Laval, Canada). The propagating strain *S. aureus* RN4220 ∆*tarM* was freshly grown in TSB to *OD*_600_ of 0.4. Afterward, the lysate containing phage P68 was added to achieve the input ratio (phage-to-bacteria ratio; IR or MOI_INPUT_) of ~ 10 and allowed to propagate at 37 °C with mild shaking until the bacterial lysis occurred. The resulting bacterial lysate was filtered through a 0.45-μm polyethersulfone syringe filter (TPP, Switzerland), and the sterile lysate was stored at 4 °C for further processing.

The phage was purified by centrifugation at 64,000 g for 2.5 h at 4 °C (Avanti J-30I with JA-30.50 Ti rotor, Beckman Coulter, USA). The pellet was resuspended in 300 µL of phage buffer overnight at 4 °C. The residual dissolved proteins were removed by extraction with chloroform (1:1 of total sample volume). The viral particles were purified by ultracentrifugation in the CsCl gradient, as described previously^43^. The viable virus particles were quantified by double agar layer plaque assay as plaque-forming units (PFU) per mL.

### Bacterial lysis monitored by turbidimetry

The lysis experiments in solution were performed in non-binding 96-well polystyrene microtiter plates (Greiner, Austria), utilizing the UPCON S-Pro reader (Labrox, Finland). The lysis was evaluated turbidimetrically as the decrease of *OD*_600_ using strains *S. aureus* RN4220 and *S. aureus* RN4220 Δ*tarM*. The bacterial culture was diluted in a TSB medium, and varying amounts of lysis agents (either lysostaphin or phage P68 in TSB) were added, always in a volume ratio of 1:4. The lysis was monitored for 10 h at 37 °C. The lysostaphin-mediated lysis was tested using the resulting concentrations of 0, 1, 10, and 100 µg/mL, with *S. aureus* diluted to *OD*_600_ of ~ 0.9 (2 × 10^8^ CFU/mL) using a TSB medium. In the case of phage-mediated lysis, the MOI_INPUT_ levels of 0, 0.01, 0.1, and 1 were tested.

In the study of PAS, two laboratory *S. aureus* strains (RN4220 and RN4220 Δ*tarM*) and two clinical strains (13/791 and 10/234) were tested to evaluate the susceptibility and resistance of each phage-antibiotic combination. Bacterial cultures (75 µL) were inoculated in the TSB medium, and 75 µL of phage P68 in the TSB medium with a resulting MOI_INPUT_ of 1 or 10 was added. Then, 50 µL of AMO, diluted in distilled water to a final concentration of 0.25 mg/L for laboratory strains and 32 mg/L for clinical strains, was added. The lysis in the case of PAS was monitored for 15 h at 37 °C.

The subinhibitory concentration of AMO was determined for all bacterial strains tested as follows: Bacterial inoculum was prepared by diluting the overnight culture with TSB to a final *OD*_600_ of ~ 0.25. In each well, 150 µL of this inoculum was mixed with 50 µL of AMO solutions, prepared by two-fold serial dilution, with the highest concentration of 32 mg/L, and the lowest one of 0.01 mg/L. Bacterial growth control and medium sterility control were included. The plate was incubated at 37 °C for 20 h with a 10-min measurement interval at 600 nm in an Infinite M200 microplate reader (Tecan, Switzerland). The minimum inhibitory concentration (MIC) was evaluated as the concentration with no signs of bacterial growth. Similar to what has been described previously^50^, we defined *MIC*_50_ of the 18-h *OD*_600_ values as the lowest AMO concentration that inhibited equal or more than 50% of the bacterial growth determined by bacterial control.

### Optimization of immobilization and monitoring of bacterial lysis using QCM

All QCM measurements were performed using the OpenQCM device (Novaetech, Italy). First, bacteria immobilization on the gold surface of 10 MHz QCM sensors was optimized. The polished and unpolished surfaces, each activated or non-activated, were compared. Each sensor was first cleaned in acetone (2 × 10 min) and isopropanol (1 × 15 min) in the ultrasonic bath. Then, the sensor was washed with distilled water and dried with compressed air. The activation of gold was performed by 500 μL of cysteamine hydrochloride (20 μg/mL) in distilled water for 2 h, followed by 500 μL of 5% glutaraldehyde in PBS for 1 h at room temperature. The sensor was then washed, inserted into the measuring cell, and the binding of PLL (10 μg/mL) was carried out at the flow rate of 20 μL/min for 20 min. Alternatively, direct binding of PLL was performed in 500 μL droplets for 1 h utilizing the same concentration. Finally, the bacterial culture of *S. aureus* in the TBS buffer was injected (~ 10^9^ CFU/mL) with a flow rate of 25 μL/min for 25 min.

The experiments with lysostaphin as a lytic agent took place at room temperature, with TBS as a running buffer. Lysostaphin concentrations of 0, 1, 3, 10, 30, and 100 µg/mL were injected for 35 min, followed by 1 h for monitoring the changes on the sensor surface. In the case of phages, the concentrations of 0, 10^7^, 10^8^, 10^9^, and 10^10^ PFU/mL were applied in the flow system for 10 min, with a monitoring time of 6 h from the phage addition. The TSB growth medium was used as a running buffer, and the measurement took place at 37 °C inside a heated box.

The PAS experiments were started by immobilizing the bacteria on the sensor surface, as described above. This was followed by replacing the running buffer with the TSB medium, and after 1 h, AMO in a resulting concentration of 0, 0.06, 0.125, or 0.25 mg/L was injected into the running medium. Subsequently, phage P68 with a resulting MOI_INPUT_ of 1 was also added to the running medium for ~ 2.5 h. The measurement took place at 37 °C for 15 h. The flow rate in all lysis experiments was 25 μL/min. The subinhibitory concentration of AMO for QCM measurements with the bacterial strain *S. aureus* RN4220 was determined in the same way as described for turbidimetry, but utilizing the dissipation signal.

### Verification of bacteria viability by fluorescence microscopy

After the immobilization of bacteria on the sensor surface, the cell culture was stained using the LIVE/DEAD *Bac*Light Bacterial Viability Kit according to the manufacturer’s instructions. The primary objective was to assess the relative viability of bacteria on the sensor after the immobilization without emphasizing their absolute numbers. The kit includes SYTO 9 (3.34 mM) and propidium iodide (PI; 20 mM) dyes, which intercalate into the DNA of the cell and enable evaluating viability based on their different permeability through the bacterial membrane. Both dyes were mixed in a 1:1 ratio (v/v) and diluted (3 μL of the mixture in 1 mL of TBS buffer). The solution was then injected into the measuring system, staining bacteria on the sensor surface.

The stained samples were observed using the BX41 fluorescence microscope (Olympus, Japan) with the veTEC monochromatic camera (Omegon, Germany) and a mercury-vapor lamp as an excitation source. SYTO 9 emission was observed using a fluorescein isothiocyanate filter cube (U-MWB2; excitation 475 ± 30 nm, emission > 520 nm, dichroic mirror 500 nm), and the PI was visualized using a tetramethyl rhodamine isocyanate filter cube (U-MWG2; excitation 530 ± 40 nm, emission > 590 nm, dichroic mirror 570 nm). The images were processed using the ImageJ software (National Institutes of Health, USA) and are presented with artificial colors.

### AFM visualization of the sensor surface

The evaluation of the roughness of polished and unpolished QCM sensors and the visualization of bacteria on the sensor surfaces were carried out using AFM in air. Before AFM scanning, each QCM sensor was washed with distilled water and dried with compressed air. The scanning was done using Dimension FastScan Bio (Bruker, USA) in PeakForce Tapping mode. A ScanAsyst-Air probe (Bruker, USA) with a spring constant of 0.4 N/m was used. The 10 µm × 10 µm images were recorded at the resolution of 1024 px × 1024 px. The images were processed in the Gwyddion software (Czech Metrology Institute, Czech Republic)^51^.

### Statistical data evaluation

The statistical evaluation was performed in OriginPro 2022 (OriginLab, USA). All turbidimetric measurements were performed in three technical replicates. Assessment of the bacteria immobilization on the sensor activated by PLL was performed five times for both the polished and the unpolished sensors. QCM measurements were repeated in two (phage- and lysostaphin-mediated lysis) or three (PAS) biological replicates, depending on the complexity and length of the measurement. Bar graphs were plotted as mean values, with error bars representing the standard deviations. In dot charts, each point corresponds to one measurement. The data was normalized according to the formula:

$${\Delta D}_{{\text{normalized}}}=\frac{\Delta L\cdot {\Delta I}_{{\text{m}}}}{\Delta I\cdot {\Delta L}_{{\text{m}}}}$$

where ∆*L* is the difference in the signal before and after the lysis, ∆*I* is the difference in the signal before and after the immobilization, and ∆*L*_m_ and ∆*I*_m_ are the signal differences in the TSB medium. It must be noted that in the case of the temperature of 37 °C, ∆*L* is affected by both the lysis and the potential bacteria growth. The standard deviations were calculated considering the error propagation. The statistical evaluation of the data was carried out by a one-way ANOVA, and the statistical significances were marked with asterisks (**** for *p* < 0.0001, *** for *p* < 0.001, ** for *p* < 0.01, * for *p* < 0.05, and ns for *p* > 0.05).

In addition to the normalization method employed here, simpler processes, such as studying the lysis in buffer at room temperature, can be explained in more details as proposed by Olsson et al.^52^, quantifying the sensor coverage and layer thickness.

## Results and discussion

### Evaluation of bacterial strain and lytic agent models

The enzyme lysostaphin and phage P68 were used as model lytic agents. With lysostaphin, the complete eradication of the bacterial cultures of *S. aureus* RN4220 and *S. aureus* RN4220 Δ*tarM* was observed by turbidimetry for all the studied concentrations (1, 10, and 100 μg/mL) within 1.5 h (Figure S2A,B).

Turbidimetry was also used to select the most effective model bacteria-phage combination (MOI_INPUT_ of 0, 0.01, 0.1, and 1) for lysis monitoring. Figure S2C shows the incomplete eradication of *S. aureus* RN4220 Δ*tarM*; the lysis was observed approximately 1 h after the phage injection, corresponding with the literature^23^. On the other hand, *S. aureus* RN4220 was not eradicated by the P68 phage, even at the highest tested MOI_INPUT_ (Figure S2D). Hence, *S. aureus* RN4220 Δ*tarM* was selected as a suitable model for optimizing QCM-D experiments with the lysis induced by phage P68.

### Optimization of *S. aureus* immobilization for real-time QCM and QCM-D measurements

The preparation of the QCM-D measurement started with modifying the gold-coated QCM sensors. Two types of sensors, polished and unpolished, were compared, and their roughness was characterized by AFM (Figure S3A,B). The unpolished sensor had a more fragmented surface, with a ~ 1 µm height range, unlike the polished with a height range of ~ 10 nm. Gentle electrostatic binding of bacteria to the sensor surface via PLL was performed^23^. The adhesion of PLL to gold (adsorption) and cysteamine-glutaraldehyde-activated sensor surfaces (covalent binding) was verified, resulting in a resonance frequency decrease of 20 to 25 Hz within 10 min for both crystal types (Figure S3C). Thus, as the results of both modification approaches were similar, further experiments were carried out with the simpler adsorption-based method.

Both crystal types were compared by monitoring the *S. aureus* immobilization on QCM sensors in the TBS buffer. Figure S4A shows the frequency and dissipation changes in time for both crystal types. The introduction of running TBS buffer after the immobilization did not result in a significant decrease in the dissipation signal that would indicate the detachment of the bacteria from the surface. This is further supported by the statistical data evaluation (Figure S4B), demonstrating that the bacteria bind efficiently without detachment and the resulting signal changes have an acceptable error. The binding of bacteria to the surface of the polished sensor resulted in a resonance frequency change of − 163 ± 62 Hz, while the changes in the case of the unpolished sensor reached − 533 ± 63 Hz. The higher surface roughness of the unpolished crystal, connected with a larger surface area, increased the number of bacteria immobilized on the sensor up to threefold. Thus, for further optimizations, the unpolished crystal was chosen for its higher yield.

Maintaining the viability and metabolic activity of bacteria is necessary for the implementation of the lytic cycle of the phage, phage reproduction, and subsequent lysis of the bacterium. The effect of immobilization on viability was verified using the LIVE/DEAD *Bac*Light Bacterial Viability Kit utilizing fluorescence microscopy. Figure S5 compares bacterial viability on polystyrene Petri dishes and polished and unpolished QCM sensors functionalized with PLL. It was shown that the immobilization did not affect the viability of the bacteria; almost all bacteria on the sensor surface were observed to be alive.

### Lysostaphin as a model for QCM and QCM-D monitoring of bacterial lysis

Lysostaphin is a very effective lytic agent suitable for optimizing the QCM setup in the study of bacterial lysis. A typical lysis curve demonstrating the changes in resonance frequency and dissipation in time is shown in Figure S6A. The buffer flowed over *S. aureus*-functionalized sensors for 20 min, followed by the injection of lysostaphin (10 μg/mL), first causing a frequency decrease by 117 ± 2 Hz due to the binding of the enzyme to the surface, followed by the frequency increase by 210 ± 120 Hz, associated with the cleavage of bacterial peptidoglycan and subsequent lysis of bacteria and release of cell fragments. The dissipation curve showed exactly the opposite trend, agreeing with the expectation. The enzyme binding caused only a slight increase in dissipation of (0.05 ± 0.02) × 10^−4^, followed by a significant decrease by (1.17 ± 0.13) × 10^−4^ during the subsequent lysis. The signal changes during the lysis strongly depended on the number of immobilized bacteria. As shown in Figure S6B, when normalized by the number of immobilized cells, the frequency and dissipation changes during the lysis on polished and unpolished sensors were not significantly different (*p* > 0.05). For studying lysis with simpler enzyme-based lytic agents, such as lysostaphin, the difference between QCM and QCM-D is insignificant; lysostaphin binding and bacterial lysis were successfully detected in both cases, concluding that both the QCM and the QCM-D are suitable for measurements in a buffer environment at room temperature.

**Fig. 2:**
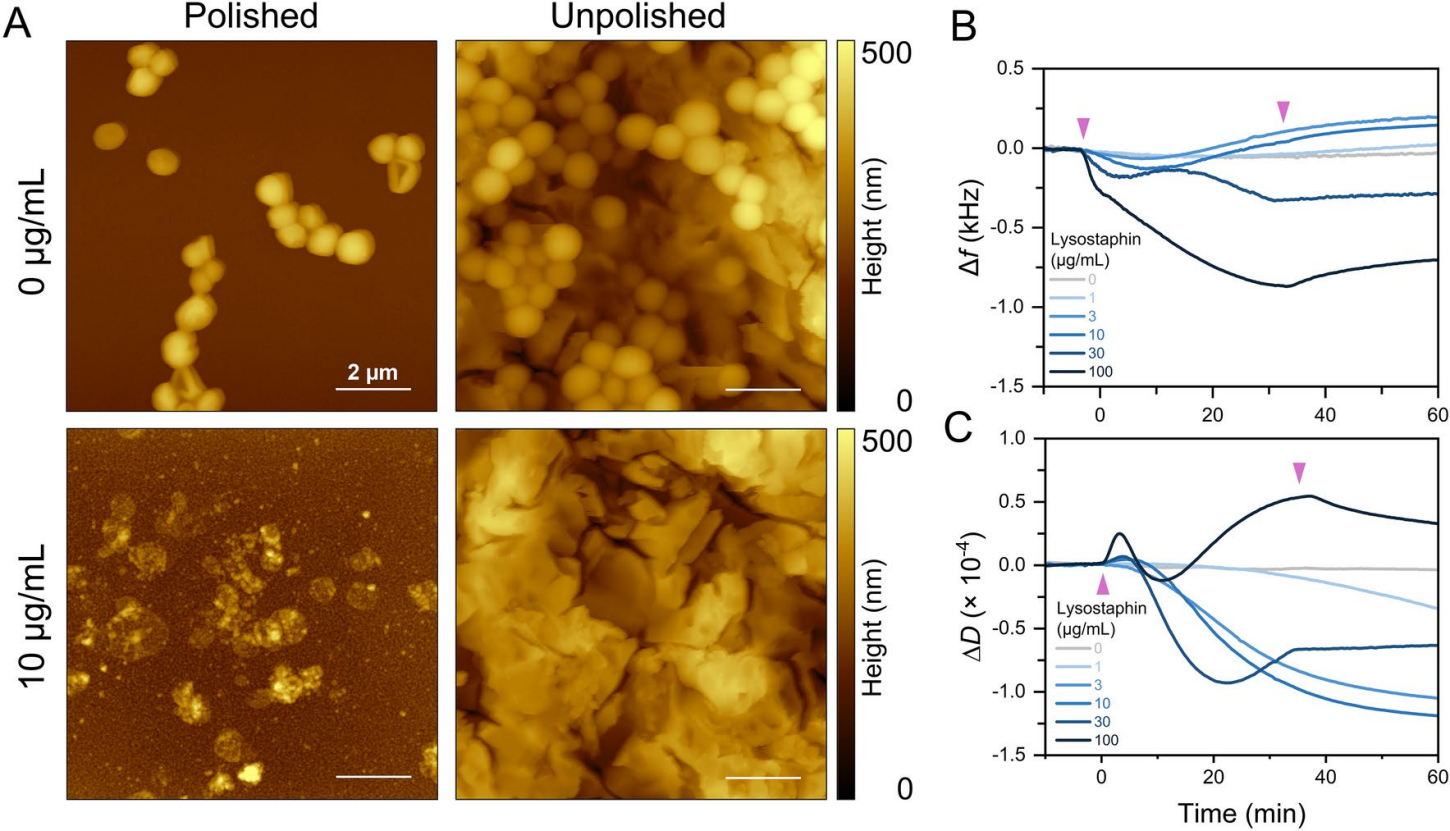
Lysostaphin-induced lysis of *S. aureus* RN4220 Δ*tarM* on polished and unpolished QCM sensors modified with PLL. (**A**) AFM visualization of QCM sensors with attached *S. aureus* without and with lysostaphin (10 μg/mL). Monitoring the changes of (**B**) resonance frequency and (**C**) dissipation of unpolished sensors during the lysis by various lysostaphin concentrations. The purple arrows indicate the start and end of the lysostaphin injection.

The AFM visualization of the sensor surfaces with attached bacteria without and with lysostaphin lysis (Fig. 2A) revealed changes in the roughness of the polished sensor after the lysis. On the other hand, with an unpolished sensor, only the absence of bacteria could be identified due to its higher roughness. Nevertheless, unpolished sensors were chosen for further experiments for their higher responses and easier handling; as the unpolished crystals are slightly thicker, better sealing of the measuring cell can be achieved, reducing the number of bubbles present throughout the experiments.

After the general optimizations, lysostaphin concentrations of 0, 1, 3, 10, 30, and 100 µg/mL were examined utilizing the unpolished crystals (Fig. 2B,C). For the low concentrations (≤ 10 µg/mL), the course of the curves was consistent with the one described above. However, at higher concentrations of 30 and 100 μg/mL, the lysis was followed by a further decrease in frequency and an increase in dissipation that lasted until the end of the lysostaphin injection. In a reference experiment with lysostaphin adsorbing to the sensor surface, the frequency and dissipation changes were significantly lower (Figure S7). Thus, the binding of bacterial residues to the sensor surface is the probable explanation for the signal changes in the later phases of the bacterium-lysostaphin interactions. It should be noted that sensor surface blocking was not carried out in these experiments. However, similar behavior for higher enzyme concentrations was previously observed also using SPR with the surface blocked using 1% bovine serum albumin^23^. The choice of a particular blocking agent can be a complex problem and should be addressed in further studies. For example, antifouling surfaces with specifically attached cells can be considered^53^.

### QCM and QCM-D monitoring of phage-mediated lysis

**Fig. 3:**
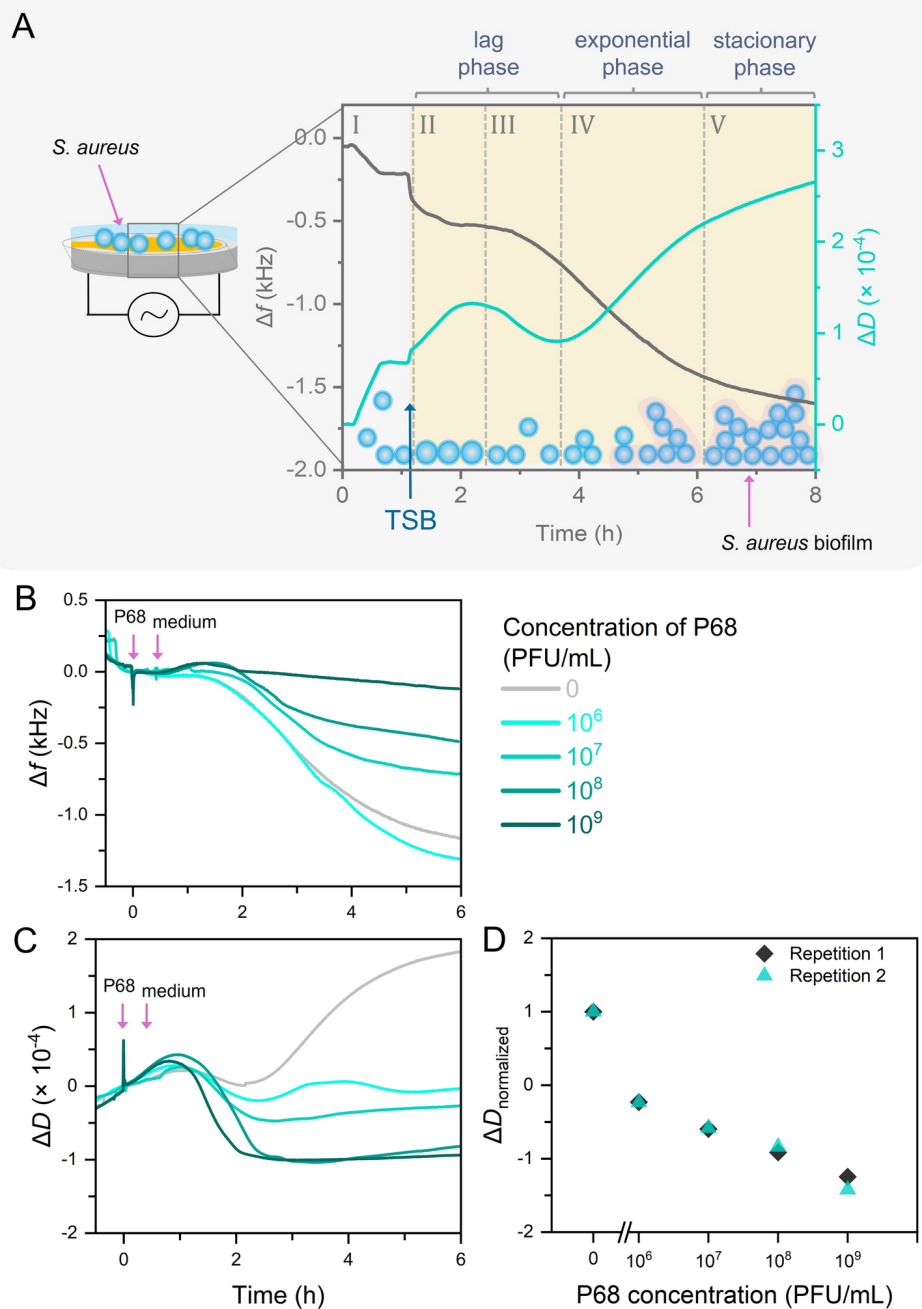
QCM-D monitoring of *S. aureus* RN4220 Δ*tarM* on the surface of the piezoelectric sensor modified with PLL. (**A**) Growth of *S. aureus* without the presence of lytic agent. Changes in (**B**) frequency and (**C**) dissipation in real-time monitoring of lysis by various concentrations of phage P68. (**D**) Dissipation data (differences between the maximum dissipation value reached after the phage addition and the minimum value) for individual phage concentrations normalized to the number of immobilized bacteria.

To carry out phage-mediated lysis, it is necessary to ensure optimal conditions for virus multiplication and the completion of the lytic cycle. Thus, the experiments were conducted at an elevated temperature of 37 °C, with the TSB growth medium as a running buffer. A typical *S. aureus* growth curve without lysis is shown in Fig. 3A. First, the immobilization of bacteria took place in the TBS buffer for greater yield and better adhesion of the cells to the surface (phase I). Next, an injection of TSB growth medium was carried out. When TSB is injected into the measuring cell, binding of proteins from the medium on the sensor surface can occur. During the medium injection, the dissipation increased by (0.135 ± 0.019) × 10^−4^, and the resonance frequency decreased by 215 ± 27 Hz within 5 min. This effect was utilized to block the sensor surface, limiting non-specific interactions of lysis agents with PLL.

The dissipation continued to increase and reached a maximum of 1 h after the TSB addition (phase II). According to Liyun Wang et al., who investigated the behavior of a bacterial culture on the polyethylene terephthalate surface using fluorescence microscopy, bacteria on a solid surface can increase their volume through metabolic processes and nutrient uptake^54^. In our study, the increase in dissipation was likely caused by such processes along with cell growth. After that, a decrease in dissipation signal was observed, which could be associated with the release of reversibly attached cells from the surface of the sensor or a reduction in the number of bacteria due to their spontaneous death (phase III). This was followed by bacterial growth, primarily in the form of biofilm, which involves bacterial division and extracellular matrix production (phase IV). As demonstrated by the increasing dissipation, the viscosity of the biofilm kept increasing until the stationary phase was finally reached (phase V). In comparison, monitoring the frequency provides less information because the difference between the binding and the lysis cannot be distinguished. This highlights the importance of monitoring dissipation in QCM-D for the study of phage-mediated lysis. The information on the changes in the character of the bacterial culture and the development of the biofilm on the sensor surface during phases II and III would be unclear by only monitoring the frequency signal. After the characterization of the growth curve, the bacterial culture was lysed using different bacteriophage concentrations (10^6^ to 10^9^ PFU/mL; Fig. 3B,C). The lysis was observed as a frequency decrease after a defined time shift, agreeing with the turbidimetric measurements (Figure S2). The decreasing trend in frequency during the lysis was similar to that of bacteria binding to the surface, suggesting that binding occurs in both cases; during the immobilization or growth, new bacteria attach, whereas during the lysis, remnants of lysed bacteria adhere. Conversely, the dissipation signal of the lysis showed an opposite trend compared to the binding of the bacteria, highlighting the importance of dissipation in assessing phage-mediated lysis. This effect is most pronounced when comparing the control without the phage with the lowest phage concentration of 10^6^ PFU/mL; the dissipation clearly shows the lysis progress, whereas the frequency signal combines the growth, lysis, and the possible deposition of lysed bacterial residues.

Figure 3D shows dissipation changes normalized to the number of immobilized bacteria for individual phage concentrations, revealing a decreasing trend in the dissipation with the increasing phage concentration for both replicates. Furthermore, a reference experiment (Figure S8) demonstrated that phage binding to the sensor surface does not result in a change in frequency or dissipation to the same extent as lysostaphin, regardless of the concentration.

Thus, we have demonstrated that the combination of frequency and dissipation monitoring provides complex information on the phage lysis, distinguishing the lysis of bacteria from the adherence of their lysed remnants to the sensor surface. This represents a significant advantage compared to the observation of phage-mediated lysis by SPR^23^, which combines the effect of lysis and adherence into a single measured signal (change of refractive index).

### QCM-D monitoring of PAS

The PAS effect was observed in the phage P68 combined with AMO as a plaque enlargement on strain RN4220 Δ*tarM* and phage propagation on strain RN4220 (Figure S1). We hypothesize that the effect of AMO, which inhibits bacterial cell wall biosynthesis and repair, resulted in either more efficient phage adsorption or a delayed lysis effect^55^, leading to increased phage burst size and, thus, larger plaques. As strains expressing the accessory cell wall teichoic acid glycosyltransferase TarM are resistant to podoviruses, such as P68^47^, the effect of AMO on cell wall structure probably plays a role in the observed gain of the ability of phage P68 to infect the strain RN4220 efficiently.

To maintain the advantages of synergistic effect (selectivity and suppression of the formation of resistant mutants), the phage must be able to propagate on a given strain. This occurs either by the treatment with an antibiotic that modifies the susceptibility of the strain or by using its subinhibitory concentration, which partially inhibits the host population but does not kill, allowing the phage to propagate^56^, in some cases even more effectively than without the antibiotic^55^. Moreover, the PAS-based approach can minimize the harmful effect of antibiotics on the natural microbiota^57^. Another important aspect of the PAS effect is the ability of phages to penetrate and disrupt the biofilm to make it accessible to antibiotics^58^.

Four *S. aureus* strains (RN4220, RN4220 Δ*tarM*, 10/234, and 13/791) were subjected to individual and combination in vitro treatment to assess the synergistic effect. First, the subinhibitory concentration of AMO for *S. aureus* strain RN4220 was determined using turbidimetry (Figure S9A) and QCM (Figure S9B). The methods provided different results, showing a subinhibitory cut-off at 4 mg/L using turbidimetry and 0.06 mg/L using QCM. This can be explained by the different numbers of target bacteria and the fact that turbidimetry evaluates planktonic culture, whereas QCM studies bacteria immobilized directly on the sensor surface. A reference experiment (Figure S10) confirmed that binding of AMO to the sensor surface without bacteria does not cause a significant change in the signal, consistent with the previous experiments with lysostaphin.

Furthermore, the effect of the synergy of phage P68 with AMO on the individual *S. aureus* strains was evaluated. Turbidimetry showed complete eradication of the P68-susceptible strain RN4220 Δ*tarM* (Figure S11A) and P68-resistant strain RN4220 (Figure S11B) in the combined treatment, in contrast to individual phage P68 treatment, which failed to eradicate even the susceptible strain. In the case of the clinical strain 13/791, the PAS accelerated the lysis process, although complete lysis was not achieved within the observed timeframe of 15 h (Figure S11C). Finally, no effect was observed in the highly resistant strain 10/234 (Figure S11D). Thus, due to the most pronounced synergistic effect, *S. aureus* RN4220 was used for further lysis monitoring experiments.

**Fig. 4:**
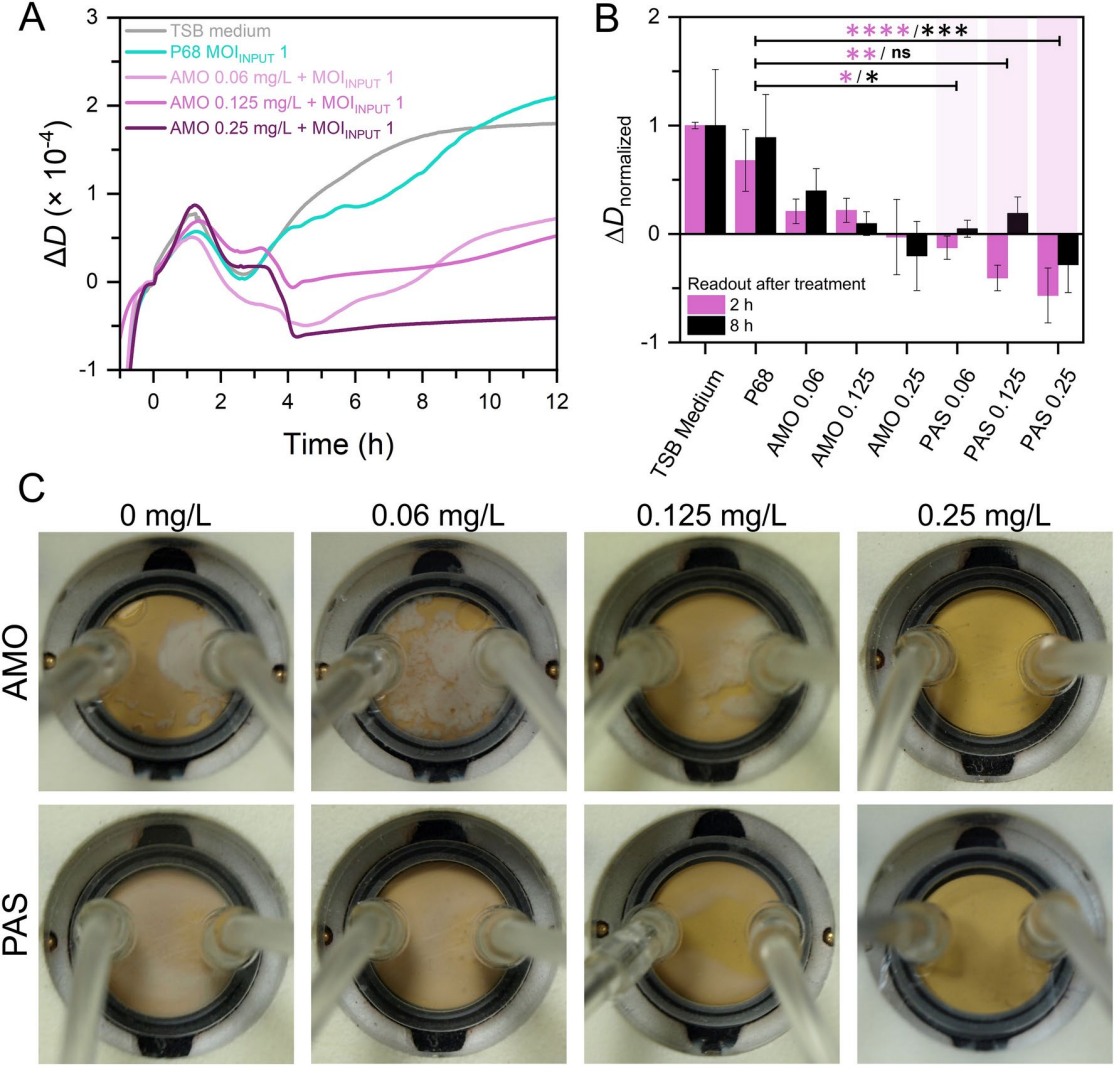
(**A**) Changes in QCM-D dissipation signal over time for the lysis of *S. aureus* RN4220 by phage P68 (MOI_INPUT_ = 1) alone and in combination with AMO (0.06 mg/L, 0.125 mg/mL, and 0.25 mg/L). (**B**) Normalized dissipation changes read out at 2 h and 8 h after the addition of various lytic agent combinations. Error bars represent standard deviations, and the statistical significances are marked with asterisks (**** for *p* < 0.0001, *** for *p* < 0.001, ** for *p* < 0.01, * for *p* < 0.05, and ns for *p* > 0.05). (**C**) The appearance of the QCM-D measuring cell after 24 h of measurement for AMO alone (concentrations provided above the corresponding panels) and PAS (utilizing 10^7^ PFU/mL of phage P68), showing the differences in the constitution of the formed bacterial biofilm (white layer). In the case of PAS, a compact biofilm was not formed.

Figure 4A shows changes in dissipation over time for the lysis of P68-resistant *S. aureus* RN4220 using the phage P68 (MOI_INPUT_ = 1) alone and in combination with AMO (concentrations 0.06, 0.125, and 0.25 mg/L). When using the phage P68 alone, the trend of the dissipation curve remained comparable to the case of negative control in the TSB medium. In the combined treatment experiment, no significant difference was observed compared to the control in the dissipation trend during the antibiotic injection step (time between 1–2 h) with all the studied AMO concentrations. However, upon the subsequent addition of phage P68 (in the time of 2.5 h), the bacterial growth ceased, followed by the sharp decrease in dissipation due to the lysis after another ~ 45 min of PAS with AMO concentrations of 0.125 mg/L and 0.25 mg/L. There was no further increase in the dissipation signal, indicating no bacterial multiplication on the sensor surface. In contrast, the lowest studied AMO concentration of 0.06 mg/L did not show a significant decrease in dissipation upon phage addition, and bacterial growth persisted.

Considering the dynamics of bacterial culture growth and lysis, dissipation changes were read at two distinct time points: 2 h after the addition of phage P68, corresponding to the maximum signal decrease, and 8 h after the addition of phage P68, at the beginning of the stationary phase observed in the control sample without treatment. The two readouts are compared in Fig. 4B as normalized values. The readout after 2 h gives a significantly more pronounced decrease in the signal for the PAS treatment. On the other hand, the readout at 8 h shows a further increase in the signal, likely due to the insufficient eradication of all bacteria. It is, therefore, useful for monitoring further culture development and biofilm growth. The phage alone did not cause a significant change in the dissipation signal for neither of the readout times (*p* > 0.05). However, statistically significant differences were observed between the use of phage P68 alone and its synergistic mixture with AMO, demonstrating a significant effect of PAS on the initially P68-resistant *S. aureus* RN4220 bacterial culture. The comparison of readout at 2 h and 8 h after the phage injection showed that the readout at 2 h provided statistically more significant results for monitoring bacterial culture lysis by the synergistic effect of phage and antibiotic, making this time more suitable for data evaluation. It must be noted that assessing frequency and dissipation changes is not the only way of data evaluation. Kinetic parameters of the interactions can also be evaluated, as proposed by Rajaram et al.^59^.

In addition, photos of the sensors were taken 24 h after the measurement (Fig. 4C). Using an ineffective AMO concentration of 0.06 mg/L resulted in a biofilm with a similar appearance to the control with no lytic agent. Despite increasing the flow rate to 44 mL/min, the biofilm remained intact and could not be detached from the sensor surface. Conversely, in all synergistic combinations, the remnants of the bacterial culture were washed away by the high flow, and no compact biofilm was formed, even at the lowest AMO concentration. Finally, QCM was used to compare the lysis of the P68-resistant strain RN4220 with the susceptible strain RN4220 Δ*tarM* (Figure S12). Unlike the injection of phage P68 alone, the combination of phage and subinhibitory AMO concentrations resulted in significant bacterial lysis and a substantial dissipation decrease within ~ 45 min for both strains.

Overall, these results agree with observations by turbidimetry (Figure S11) and provide insights into bacterial behavior during lysis on the surface, where bacteria commonly proliferate and pose significant clinical challenges. Furthermore, the ability to monitor bacterial growth and lysis in a moderate flow can facilitate the addition of lytic agents and fresh nutrients and the collection of metabolites or cells at various growth and lysis phases for further complementary studies, such as investigating the presence of signaling molecules and genetic changes.

It should be noted that the current literature lacks comprehensive studies on bacterial behavior and lysis on surfaces toward creating robust *pathogen-on-a-chip* models. When developing such models, emphasis should be placed on scenarios resembling real-life situations where bacteria colonize mucous membranes and tissues and grow resistant biofilms. Appropriate consideration of surrounding conditions, such as body temperature, nutrient presence, and the inherent interactions between bacteria and phages, is essential. This proof-of-concept study showed that label-free QCM-D biosensors can address these gaps and provide critical insights into bacterial lysis mechanisms.

QCM-D is an affordable device with the possibility of multi-channel measurement and automation of the entire process. As the price of sensors is low (QCM sensors with Au electrodes can be obtained for €5 per piece), they can be disposed of after the measurement, not risking potential contamination due to their cleaning and reuse. In addition, both sides of the resonator can be used for experiments, further reducing the price per experiment. After optimization and appropriate technical implementation, the method can be used in clinical practice to rapidly determine the effect of lytic agents (phages, antibiotics, or other antimicrobials) or to determine whether the phage-antibiotic combination exhibits the desired efficiency, minimizing the requirement for time-consuming culture experiments.

## Conclusions

In this study, we report on a new label-free biosensor concept for sensitive real-time monitoring of bacterial lysis in a microfluidic chip. We demonstrate that by monitoring dissipation changes during lysis reactions by means of quartz crystal microbalance technique, it is possible to distinguish between lysis by cell-attached agent and other processes, such as bacteria culture growth. *S. aureus* lysis mechanisms, induced by both enzyme lysostaphin and phage P68, were investigated as model complex biological systems. Our investigations uncovered distinct mechanisms of bacterial lysis, both enzyme- and phage-mediated, shedding light on previously unexplored dynamics under viable culture conditions. Notably, we observed a unique pattern of phage-mediated lysis on a solid surface, involving bacterial growth and detachment from surfaces, a phenomenon with important implications for understanding biofilm dynamics and antibiotic resistance. Furthermore, our study revealed the potential of phage-antibiotic synergy in lysing even non-susceptible *S. aureus* strains. This finding underscores the promise of our approach in tackling infections caused by multidrug-resistant bacterial strains and biofilms, offering a significant contribution to the field of antimicrobial therapy.

## Supplementary Information

Supplementary Information.

## Data Availability

The datasets generated during the study are available from the corresponding author on reasonable request.
