# Supplementary material for: Piezoelectric biosensor with dissipation monitoring enables the analysis of bacterial lytic agent activity
Source: Sci Rep. 2025 Jan 27;15:3419. doi: 10.1038/s41598-024-85064-x (PMC11772602; doi:10.1038/s41598-024-85064-x)
Supplement: Supplementary file 1 — Supplementary Information. [file 41598_2024_85064_MOESM1_ESM.pdf]

SUPPLEMENTARY INFORMATION
Piezoelectric biosensor with dissipation monitoring enables the analysis of bacterial lytic agent activity

Radka Obořilová[1,2,*], Eliška Kučerová[3], Tibor Botka[3], Hana Vaisocherová-Lísalová[4], Petr Skládal[1], and Zdeněk Farka[1,2,*]

[1] *Department of Biochemistry, Faculty of Science, Masaryk University, Kamenice 5, 625 00 Brno, Czech Republic*

[2] *Central European Institute of Technology, Masaryk University, Kamenice 5, 625 00 Brno, Czech Republic*

[3] *Department of Experimental Biology, Section of Genetics and Molecular Biology, Faculty of Science, Masaryk University, Kamenice 5, 625 00 Brno, Czech Republic*

[4] *Institute of Physics of the Czech Academy of Sciences, Na Slovance 1999/2, 182 21 Prague, Czech Republic*

* Corresponding author: radka.oborilova@ceitec.muni.cz (R.O.), farka@mail.muni.cz (Z.F.)

Table of contents

Figure S1: P68 propagation on RN4220 and RN4220Δ*tarM* in the presence of AMO S-2

Figure S2: Lysostaphin- and phage-mediated bacterial lysis monitored by turbidimetry S-2

Figure S3: Comparison of QCM sensor surfaces S-3

Figure S4: Effect of sensor surface roughness on the immobilization efficiency S-3

Figure S5: Viability of *S. aureus* cells immobilized on the surface S-4

Figure S6: Lysis efficiency for different sensor surfaces S-4

Figure S7: Binding of lysostaphin to the QCM sensor surface S-5

Figure S8: Binding of phage P68 to the QCM sensor surface S-5

Figure S9: Determination of the sub-inhibitory concentrations of amoxicillin S-6

Figure S10: Binding of amoxicillin to the QCM sensor surface S-6

Figure S11: Phage-antibiotic synergy monitored using turbidimetry S-7

Figure S12: Comparison of synergy effect on *S. aureus* RN4220 and RN4220 Δ*tarM* S-8

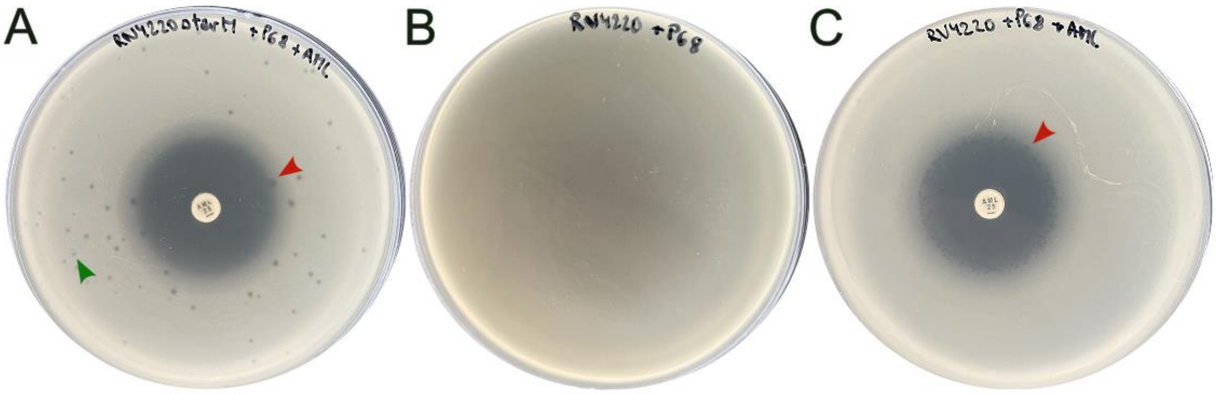

Figure S1: (**A**) *S. aureus* RN4220 Δ*tarM* infected by phage P68 on a dish with amoxicillin disc. The green arrow shows normal phage plaque, and the red arrow shows enlarged plaque. *S. aureus* RN4220 infected by phage P68 on dishes (**B**) without and (**C**) with amoxicillin disc. Compared to the culture without amoxicillin, many plaques appeared in the inhibition zone of amoxicillin.

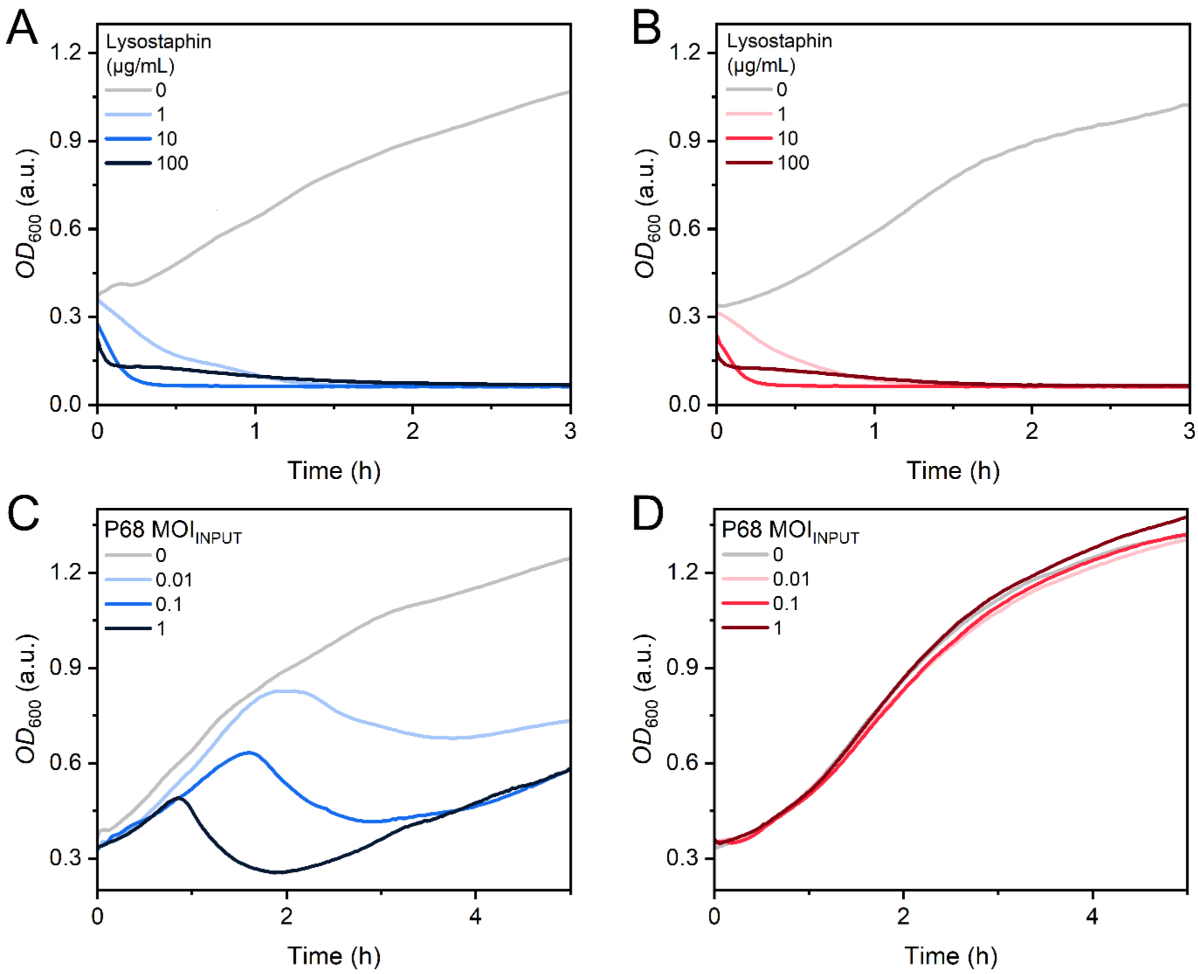

Figure S2: Bacterial lysis monitored by turbidimetry at 37 °C in TSB medium. Lysis of laboratory strains (**A**) *S. aureus* RN4220 Δ*tarM* and (**B**) *S. aureus* RN4220 mediated by the enzyme lysostaphin. Lysis of (**C**) *S. aureus* RN4220 Δ*tarM* and (**D**) *S. aureus* RN4220 by the lytic phage P68.

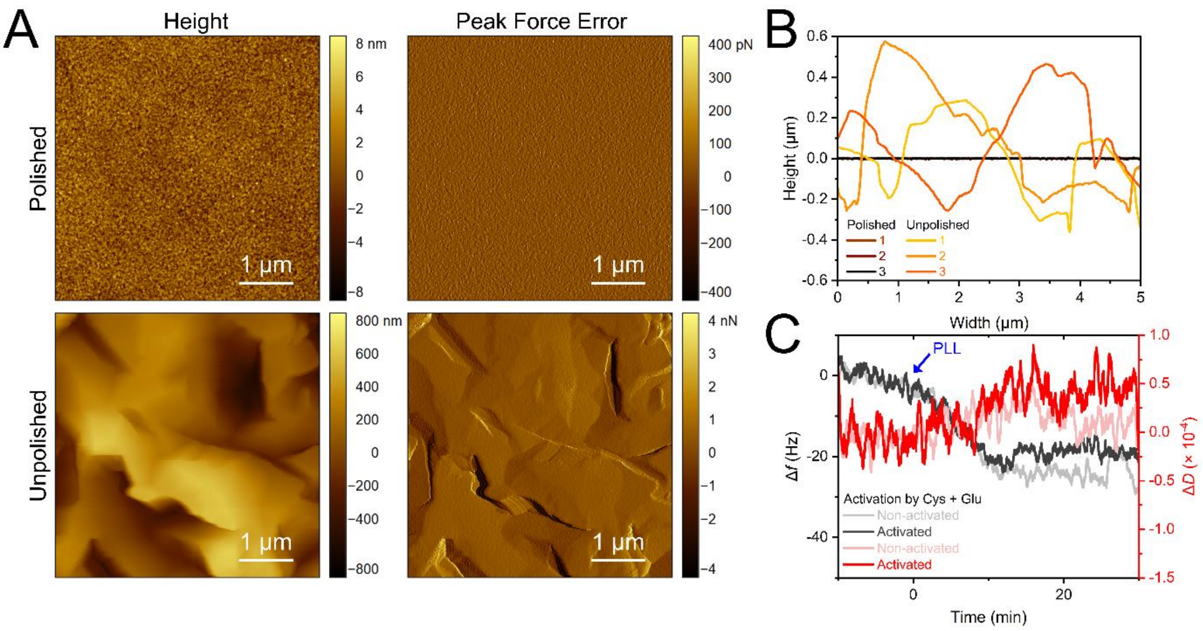

Figure S3: AFM characterization of polished and unpolished QCM sensors. (**A**) Visualization in height and peak force error signals and (**B**) representative cross-sections of height profiles. (**C**) QCM measurement of binding of poly-L-lysine (10 µg/mL) to non-activated and cysteamine- and glutaraldehyde-activated surface of the unpolished sensor.

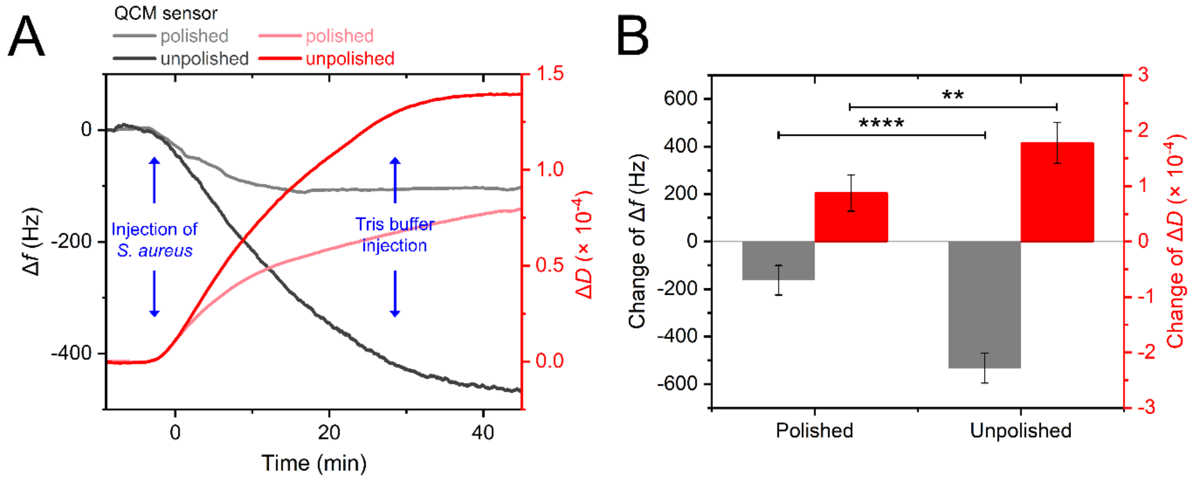

Figure S4: Effect of sensor surface roughness on the efficiency of bacteria immobilization. Changes of resonance frequency and dissipation (**A**) in time and (**B**) after the immobilization of *S. aureus* RN4220 Δ*tarM* (~ 10^9 CFU/mL) to the PLL-modified surface of the polished and unpolished QCM sensor. Significant differences in bacteria immobilization levels on polished and unpolished sensors were observed in the frequency ($p < 0.0001$) as well as the dissipation signal ($p = 0.0086$). Error bars represent standard deviations.

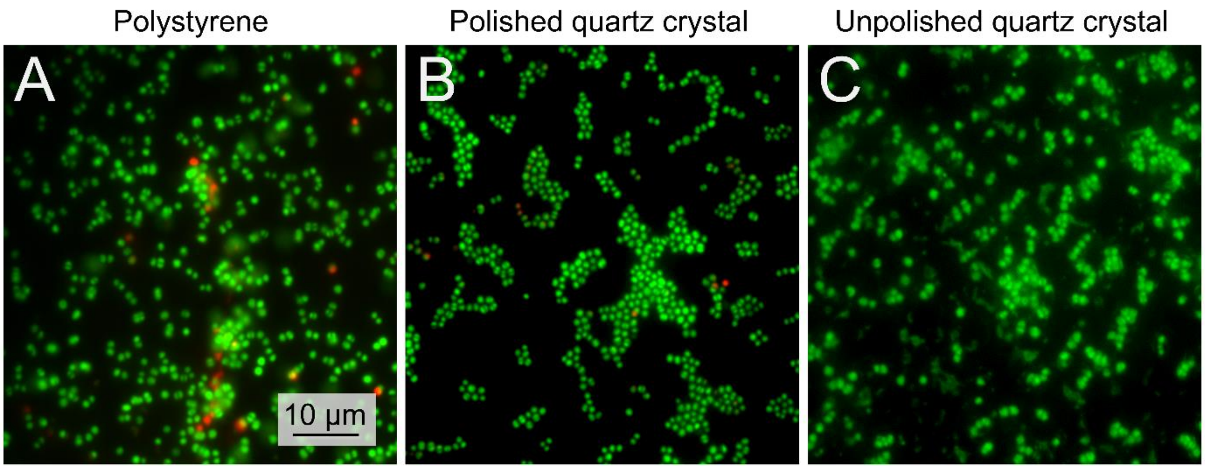

Figure S5: Viability of *S. aureus* RN4220 Δ*tarM* cells visualized using LIVE/DEAD staining kit and fluorescence microscopy. Bacteria immobilized on the surface of (**A**) polystyrene Petri dish and PLL-modified (**B**) polished and (**C**) unpolished QCM sensor. The binding to the QCM sensor was carried out online in the measuring system. Green cells are live and red cells are dead.

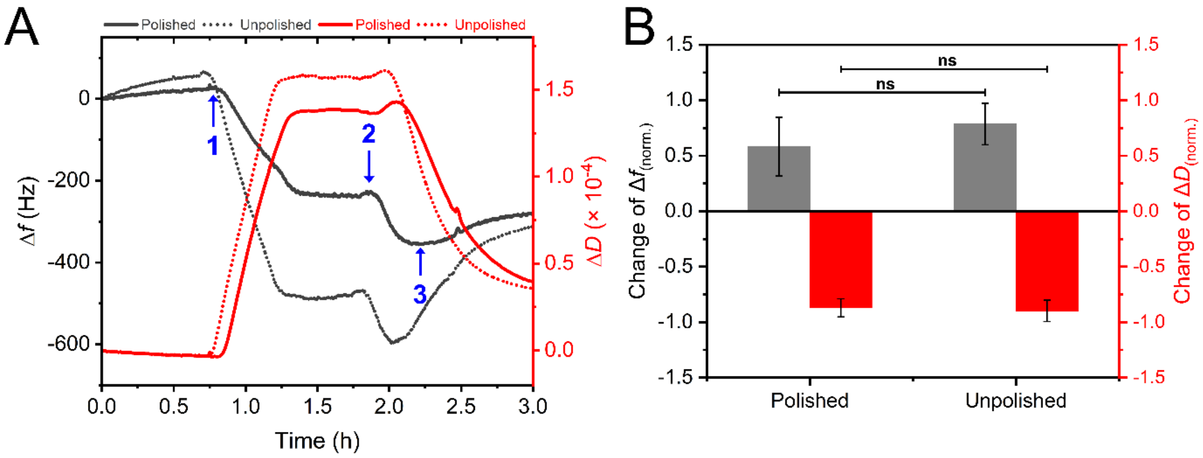

Figure S6: (**A**) Typical QCM-D curve of the lysis of *S. aureus* RN4220 Δ*tarM* by lysostaphin, showing the changes of resonance frequency and dissipation in time for polished and unpolished sensors: (1) immobilization of bacteria, (2) binding of lysostaphin, and (3) lysis of the bacteria. (**B**) The results of the lysis normalized to the number of immobilized bacteria. The differences in frequency and dissipation changes during the lysis on polished and unpolished surfaces were not significantly different ($p > 0.05$). Error bars represent standard deviations.

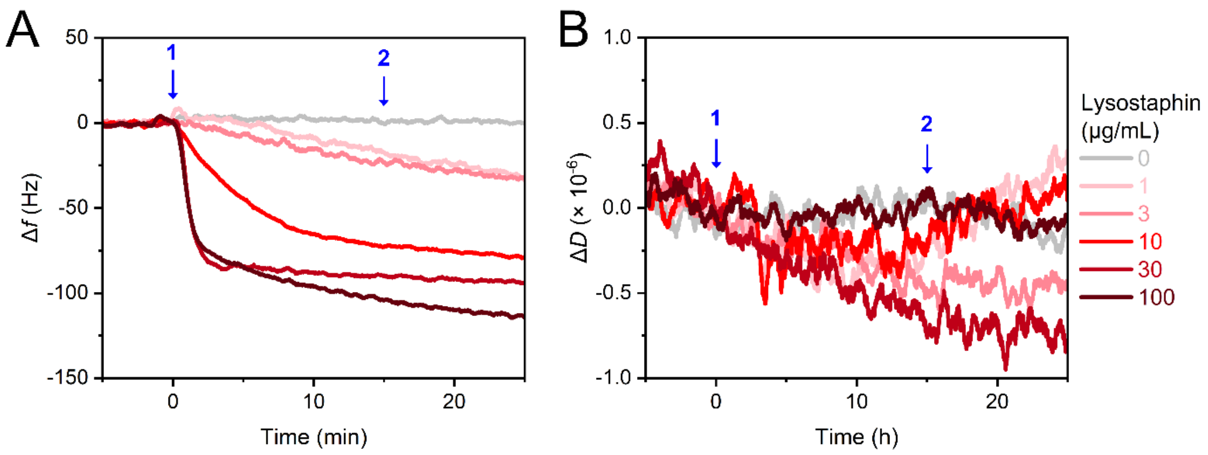

Figure S7: Binding of lysostaphin to the QCM sensor surface measured as changes of (**A**) resonance frequency and (**B**) dissipation at room temperature in TBS buffer. The blue arrows indicate (1) the start and (2) the end of the lysostaphin injection.

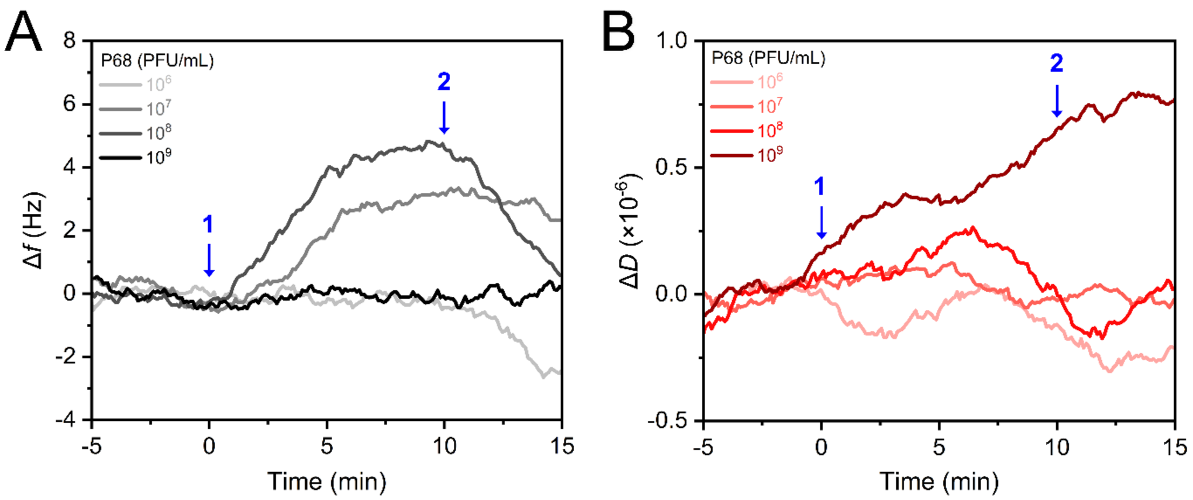

Figure S8: Binding of phage P68 to the QCM sensor surface measured as changes of (**A**) resonance frequency and (**B**) dissipation at 37 °C in TSB medium. The blue arrows indicate (1) the start and (2) the end of phage injection.

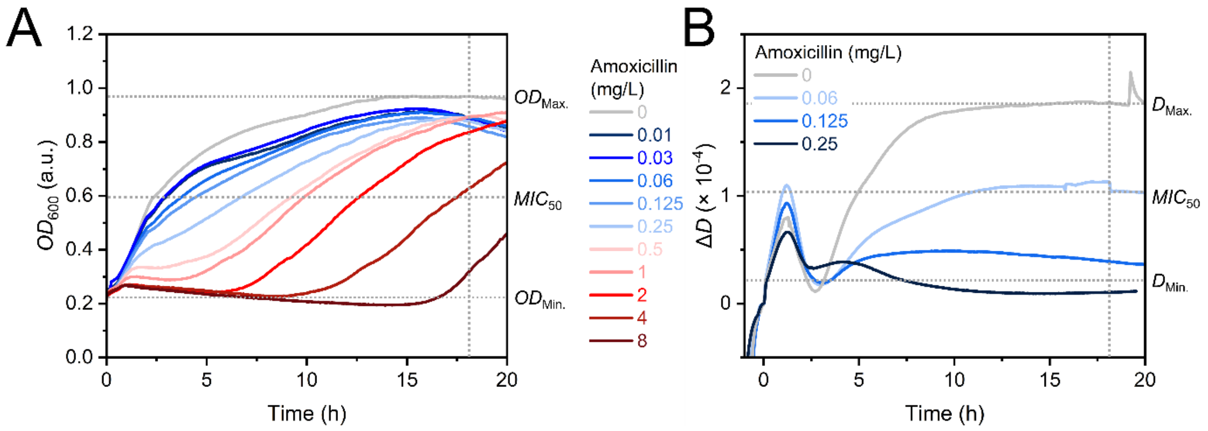

Figure S9: Determination of sub-inhibitory concentrations of amoxicillin for *S. aureus* RN4220 (**A**) in solution using turbidimetry and (**B**) on the surface utilizing QCM-D. The grey dashed line indicates the MIC_{50} criterion, i.e., 50% bacterial growth after 18 h of incubation relative to the growth control.

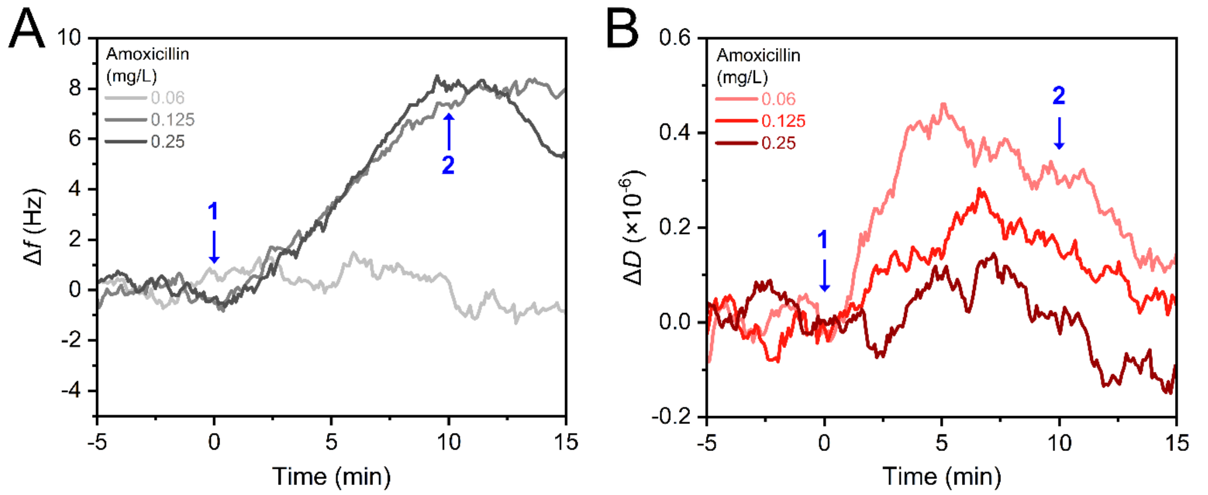

Figure S10: Binding of amoxicillin to the QCM sensor surface measured as changes of (**A**) resonance frequency and (**B**) dissipation at 37 °C in TSB medium. The blue arrows indicate (1) the start and (2) the end of amoxicillin injection.

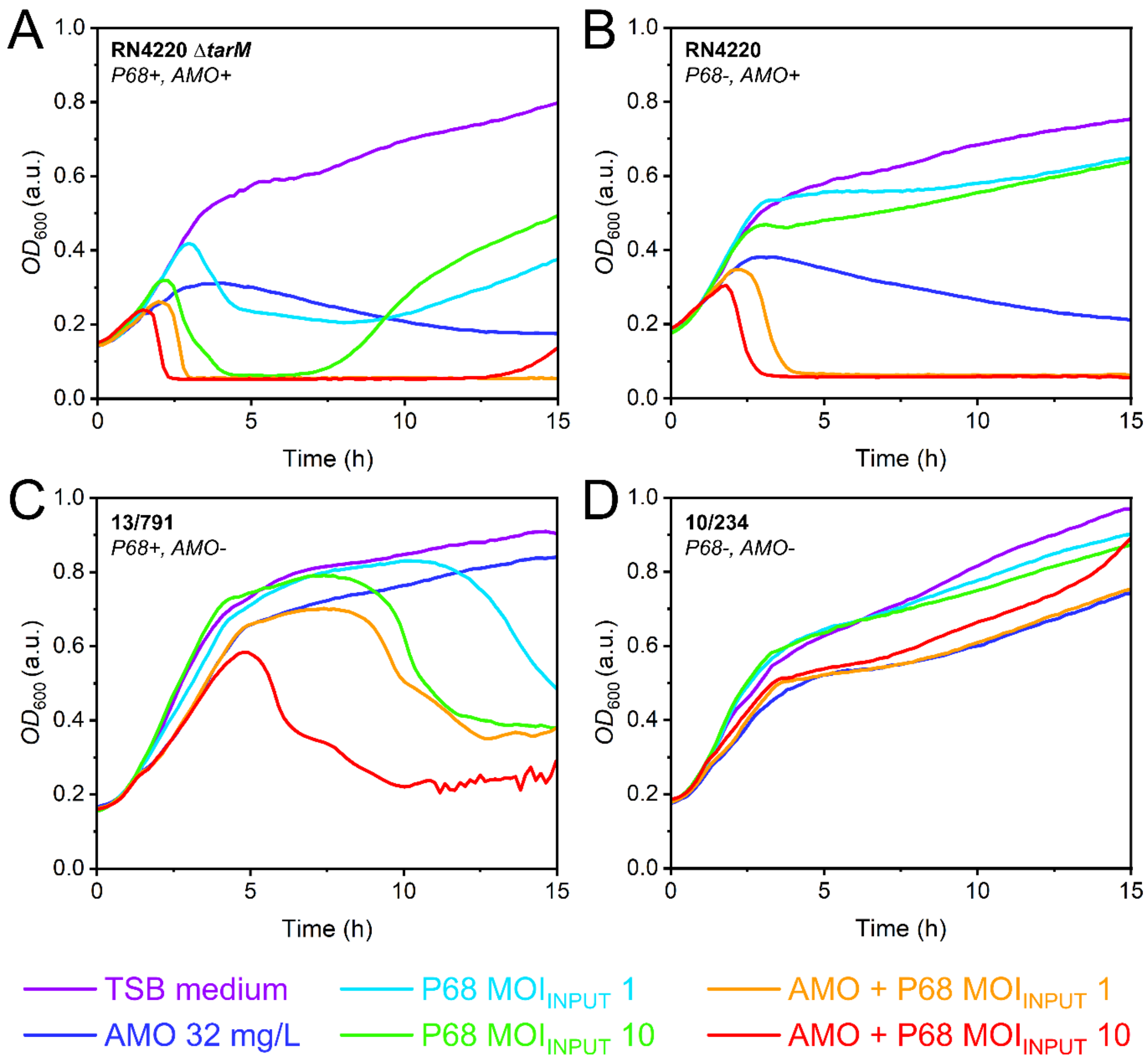

Figure S11: Turbidimetric monitoring of the lysis of *S. aureus* strains (**A**) RN4220, (**B**) RN4220 Δ*tarM*, (**C**) 13/791, and (**D**) 10/234. The studied amoxicillin (AMO) concentration was 0.25 mg/L for strains RN4220 and RN4220 Δ*tarM* and 32 mg/L for clinical strains 13/791 and 10/234. The sign + corresponds to *sensitivity* and − to resistance of the bacterium to the given lytic agent.

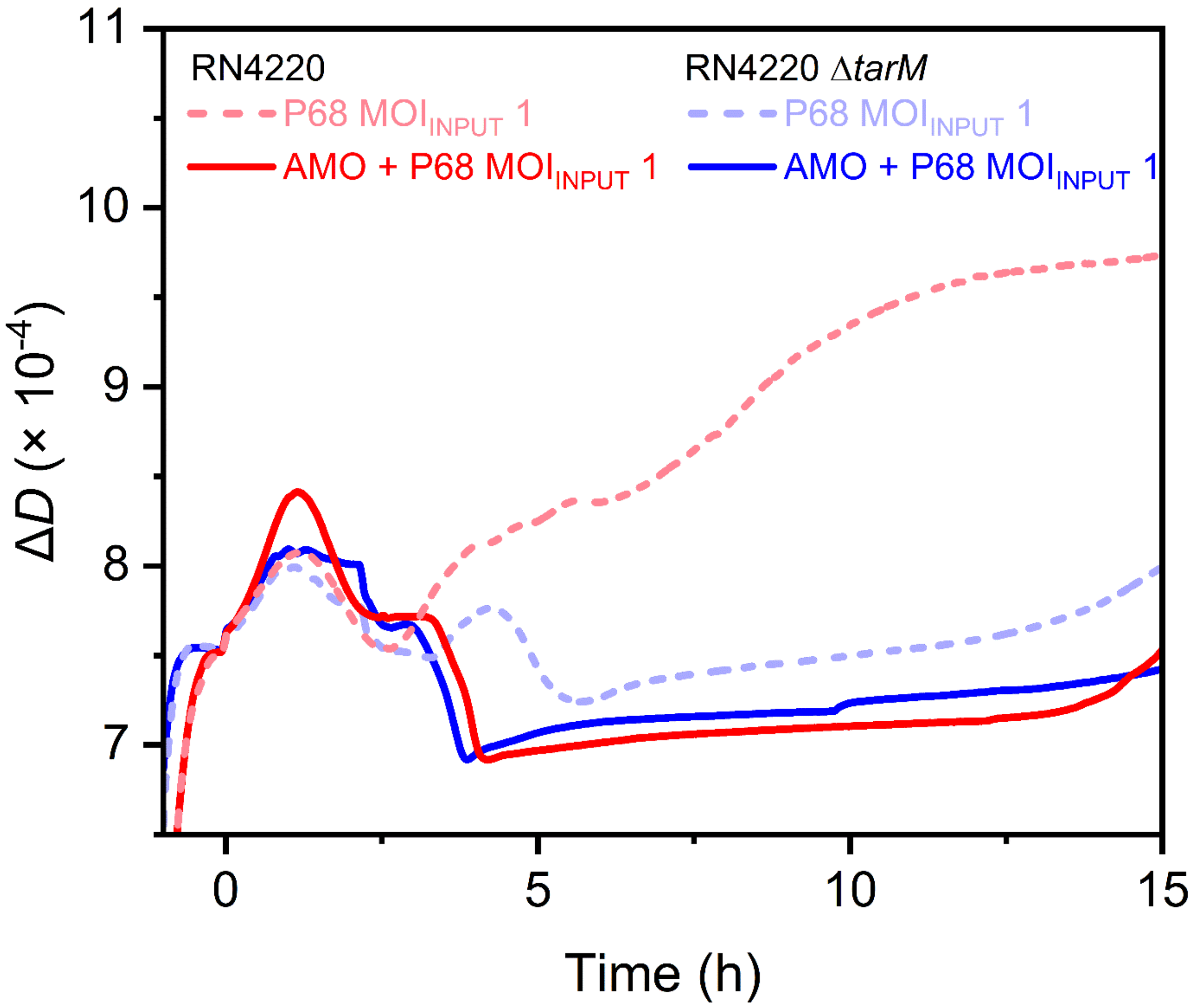

Figure S12: QCM-D comparison of the effect of phage P68 and combined phage-antibiotic treatment (AMO + P68) on phage P68-susceptible *S. aureus* strain RN4220 Δ*tarM* and P68-resistant strain RN4220. The AMO concentration was 0.25 mg/L.
